# CHALLENGES AND KEY ASPECTS OF COLLABORATION BETWEEN VISITING NURSES AND CARE MANAGERS IN JAPAN

**DOI:** 10.1093/geroni/igae098.2384

**Published:** 2024-12-31

**Authors:** Keiko Ono

**Affiliations:** Aomori University of Health and Welfare, Aomori-City, Aomori, Japan

## Abstract

Effective collaboration between visiting nurses and care managers is crucial for older adults requiring end-of-life care at home. This study aimed to elucidate key challenges and crucial cooperation between visiting nurses and care managers in Japan’s home-based end-of-life care, addressing the unclear collaboration status. The study was conducted between March and April 2023 at home-visit nursing stations in a Japanese prefecture. Self-administered questionnaires were mailed to visiting nurses, and 136 responses (70.8%) were received. A total of 125 questionnaires (65.1%) were analyzed. Further, visiting nurses answered all questions about problems and essential cooperation aspects. Of the 13 questions regarding challenges, the following question received the most responses (69.6%) of “I agree” or “somewhat agree.” The care manager must be consulted, and their approval must be obtained owing to limited amounts in the case of long-term care insurance, even if the urgent need for temporary visiting nursing care is recognized.” Conversely, of the 16 questions about what is essential in at-home end-of-life care, the following question received the most responses (98.4%) of “I agree” or “somewhat agree.” The question was, “It is important to respect the feelings and wishes of patients and their families regarding where and how they will die.” The second most common response (97.6%) was: “Everyone should have a comprehensive discussion, share awareness, and collaborate to achieve patient-satisfactory-care since neither the patients nor their families have any experience.” This study could contribute to improving their collaboration with each other.

